# Mutation of MtrA at the Predicted Phosphorylation Site Abrogates Its Role as a Global Regulator in Streptomyces venezuelae

**DOI:** 10.1128/spectrum.02131-21

**Published:** 2022-03-16

**Authors:** Ting Lu, Yanping Zhu, Xue Ni, Xia Zhang, Yang Liu, Xiqing Cui, Xiuhua Pang

**Affiliations:** a The State Key Laboratory of Microbial Technology, Shandong University, Qingdao, China; b Qingdao Vland Biotech Group, Inc., Qingdao, China; c Deqiang Biology Co. Ltd., Harbin, China; College of New Jersey

**Keywords:** *Streptomyces*, phosphorylation, MtrA, D53, regulation, gene regulation

## Abstract

The global regulator MtrA controls development and primary and secondary metabolism in Streptomyces species. However, residues critical for its function have not yet been characterized. In this study, we identified residue D53 as the potential phosphorylation site of MtrA from Streptomyces venezuelae, a model Streptomyces strain. MtrA variants with amino acid substitutions at the D53 site were generated, and the effects of these substitutions were evaluated *in vitro* and *in vivo*. We showed that, although substitutions at D53 did not alter MtrA’s secondary structure, the MtrA D53 protein variants lost the ability to bind known MtrA recognition sequences (MtrA sites) in electrophoretic mobility shift assays. Complementation of the Δ*mtrA* strain with MtrA D53 protein variants did not affect overall strain growth. However, in comparison to the wild-type strain, chloramphenicol and jadomycin production were aberrant in the D53 variant strains, with levels similar to the levels in the Δ*mtrA* strain. Transcriptional analysis showed that the expression patterns of genes were also similar in the Δ*mtrA* strain and the D53 variant strains. Although the D53 protein variants and wild-type MtrA were produced at similar levels in S. venezuelae, chromatin immunoprecipitation-quantitative PCR results indicated that replacing the D53 residue rendered the altered proteins unable to bind MtrA sites *in vivo*, including MtrA sites that regulate genes involved in nitrogen metabolism and in chloramphenicol and jadomycin biosynthesis. In conclusion, our study demonstrates that the predicted phosphorylation site D53 is critical for the role of MtrA in regulation and suggests that MtrA functions in a phosphorylated form in the genus Streptomyces.

**IMPORTANCE** Although phosphorylation has been shown to be essential for the activation of many response regulator proteins of two-component systems, the role of the phosphorylation site in the function of the global regulator MtrA in the genus Streptomyces has not been reported. In this study, we generated Streptomyces mutants that had amino acid substitutions at the predicted phosphorylation site of MtrA, and the effects of the substitutions were investigated by comparing the phenotypes of the resulting strains and their gene expression patterns with those of the wild-type strain and an MtrA deletion mutant. The ability of the altered proteins to bind known promoter targets *in vitro* was also evaluated. Our analyses showed that the predicted phosphorylation site D53 is critical for MtrA binding *in vitro* and for the normal functioning of MtrA *in vivo*. These studies further demonstrate the importance of MtrA as a global regulator in the genus Streptomyces.

## INTRODUCTION

Streptomyces species have a complex life cycle involving spore formation and exhibit great potential in producing antibiotics (1–3). Although they are mostly soil inhabiting, Streptomyces species have also been isolated from other environments, such as polar regions, hot springs, and other extreme niches. This broad environmental spread of members of the genus indicates an ability to adapt to a variety of conditions, which is in part due to a large number of two-component systems (TCS) encoded by Streptomyces genomes (4, 5). TCSs comprise a family of regulatory systems that sense and respond to diverse extracellular signals, enabling microbes to survive better under stress conditions (6).

MtrAB is one of the TCSs conserved in actinobacteria, including the genus Streptomyces (7). The MtrAB TCS is essential in Mycobacterium tuberculosis (8), and previous studies indicated that MtrA_TB_ (MtrA of M. tuberculosis), the response regulator of the MtrAB system, may serve as a coordinator between the proliferative and pathogenic functions of M. tuberculosis (9, 10). Although not essential in Corynebacterium glutamicum, MtrA_CG_ (MtrA of C. glutamicum) is required for maintenance of cell morphology, antibiotic susceptibility, and osmoprotection (11).

For a typical paired TCS, signal transduction involves the transfer of a phosphate group from a conserved histidine residue of the sensor kinase protein to a conserved aspartate residue of the response protein (12, 13). At least two conserved aspartate residues (D13 and D56) were identified in MtrA_TB_ (14), and one (D53) was identified for MtrA_CG_ (15). The functioning of MtrA is dependent on its ability to be phosphorylated in M. tuberculosis (9, 10, 16) and C. glutamicum (15). Both D13 and D56 are critical for phosphorylation of MtrA_TB_; however, only the replacement of D56 impaired the ability of the resulting mutants to block phagosome-lysosome fusion (10). In addition, the ratio of phosphorylated versus unphosphorylated forms of MtrA_TB_ may impact the regulation of cell division in M. tuberculosis (9). Another study indicated that the replacement of D53 of MtrA_CG_ abolished its ability to regulate target genes (15).

Preliminary systemic transposon mutagenesis revealed a role for MtrA in antibiotic biosynthesis in Streptomyces species (17), which was verified by later studies (18–20). Our group characterized additional roles for MtrA in the control of cellular development (21) and phosphate metabolism (22). Intriguingly, we found that the consensus sequence recognized by MtrA (MtrA site) is highly similar to the consensus sequence recognized by GlnR (23), which is the major nitrogen metabolism regulator and which activates nitrogen metabolism genes under nitrogen-limiting conditions (24, 25), and thus, MtrA competes with GlnR for nitrogen metabolism control (23, 26). However, in contrast to GlnR, MtrA serves as a repressor for nitrogen metabolism genes and profoundly represses nitrogen metabolism genes under nutrient-rich conditions, explaining the silencing of these genes under these conditions (23). Although it is known that MtrA is a global regulator in the genus Streptomyces, it is not known whether MtrA functions in a phosphorylated or unphosphorylated form in Streptomyces species. In this study, we identified a potential phosphorylation site of MtrA in a model species of Streptomyces and evaluated the role of the predicted site by mutation. We showed that the D53 residue is critically required for the role of MtrA, indicating that MtrA functions in a phosphorylated form in Streptomyces species.

## RESULTS

### Identification of the potential phosphorylation site for MtrA of *S. venezuelae*.

MtrA is highly conserved in actinobacteria (7), and the MtrA protein displays an overall amino acid sequence identity of 98% among Streptomyces species (http://strepdb.streptomyces.org.uk/). Notably, highly similarity was also demonstrated between MtrA of Streptomyces species and its homologues in the mycobacteria and corynebacteria (7). For example, MtrA_SVE_ (MtrA of Streptomyces venezuelae) has 69% amino acid identity and 82% similarity with MtrA_CG_ and 75% identity and 88% similarity with MtrA_TB_, indicating an important role for MtrA in actinobacteria. The structure of MtrA_TB_ has been solved, and residue D56 of this protein becomes phosphorylated at the side chain (27). To determine the potential phosphorylation site for MtrA_SVE_, the amino acid sequence was evaluated with SWISS-MODEL, using the amino acid sequence of MtrA_TB_ as the primary sequence for modeling. The aspartate residue at 53 (D53), which is at the same position as experimentally determined for MtrA_CG_ (15), was predicted to be the phosphorylation site (Fig. S1A in the supplemental material).

### Replacement of D53 does not alter the overall structural characteristics of MtrA_SVE_.

To investigate whether amino acid substitutions for D53 affected the physical characteristics of the protein, *mtrA*_SVE_ was mutated at the D53 codon, generating MtrA_SVE_ variants with alanine (D53A), glutamine (D53E), or asparagine (D53N). Modeling using the sequence of MtrA_TB_ as the template predicted that these changes would not alter the secondary structure (Fig. S1A). The heterologously expressed proteins were purified by column affinity chromatography and detected by sodium dodecyl sulfate-polyacrylamide gel electrophoresis (SDS-PAGE). The wild-type (WT) MtrA and its variants were purified as single bands corresponding to the size of MtrA plus a 6×His tag (Fig. S1B), suggesting that the various *mtrA* mutations did not affect gene expression or the solubility of the resulting D53 variants. The secondary structures of MtrA and its variants were also analyzed by circular dichroism (CD) spectroscopic assays using the purified proteins (Fig. S1C). The curves of the three altered MtrA proteins completely overlapped with that of the wild-type MtrA protein, indicating that the substitutions at D53 did not impact the secondary structure of MtrA, consistent with the modeling.

### Replacement of D53 abolishes binding activity of MtrA *in vitro*.

**FIG 1:**
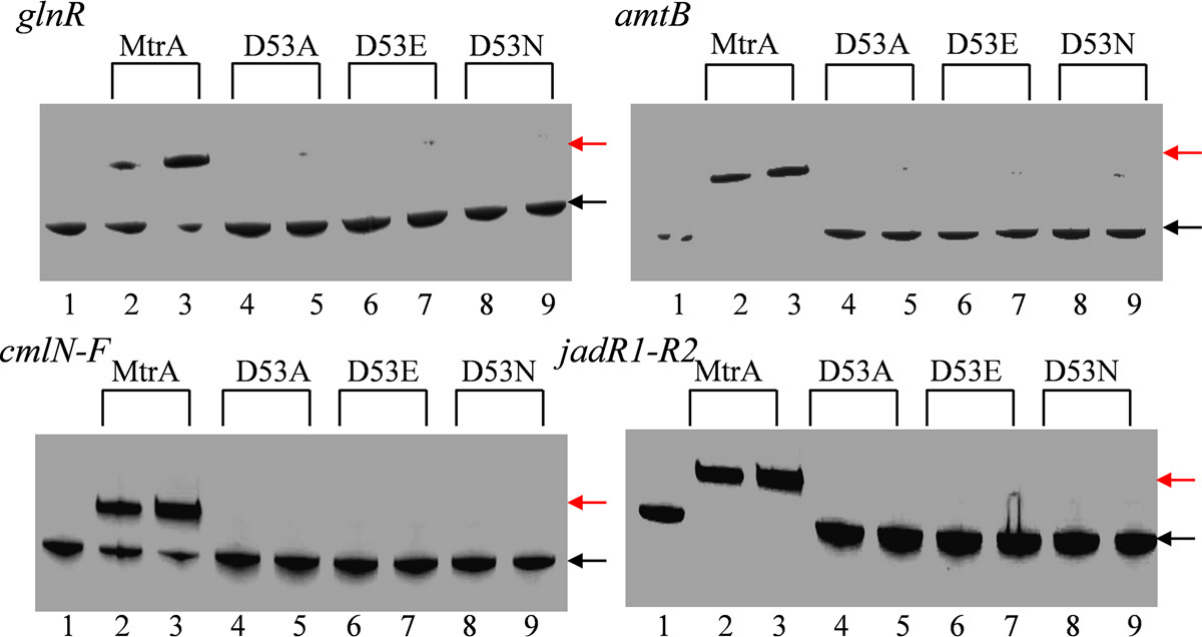
Comparison of the binding of wild-type MtrA and its variants to target sites by EMSA. Probes were used with (lanes 2 and 3) MtrA, (lanes 4 and 5) MtrAD53A, (lanes 6 and 7) MtrAD53E, or (lanes 8 and 9) MtrAD53N. Reactions were carried out with the addition of no MtrA (lanes 1) or 4.7 μM (lanes 2, 4, 6, 8) or 8.3 μM (lanes 3, 5, 7, 9) MtrA or its variants. Red and black arrows indicate the positions of the shifted and free probes, respectively.

To investigate whether the amino acid substitutions at D53 impacted the binding of MtrA to its target sites, we performed electrophoretic mobility shift assays (EMSAs) using purified wild-type protein and the altered MtrA proteins (Fig. 1 and Fig. S2). As expected, the wild-type MtrA bound the MtrA sites for nitrogen metabolism genes, such as *amtB*, *glnR*, *glnII*, and *glnA*, and genes for antibiotic biosynthesis, such as *cmlN-1* and *jadR1-R2*, in agreement with a previous report (18). In contrast, no shifting or only very weak shifting of these probes was detected with any of the altered forms of MtrA (MtrAD53A, MtrAD53E, or MtrAD53N), indicating that these amino acid substitutions abolished MtrA binding to the target sites under the conditions tested.

**FIG 2:**
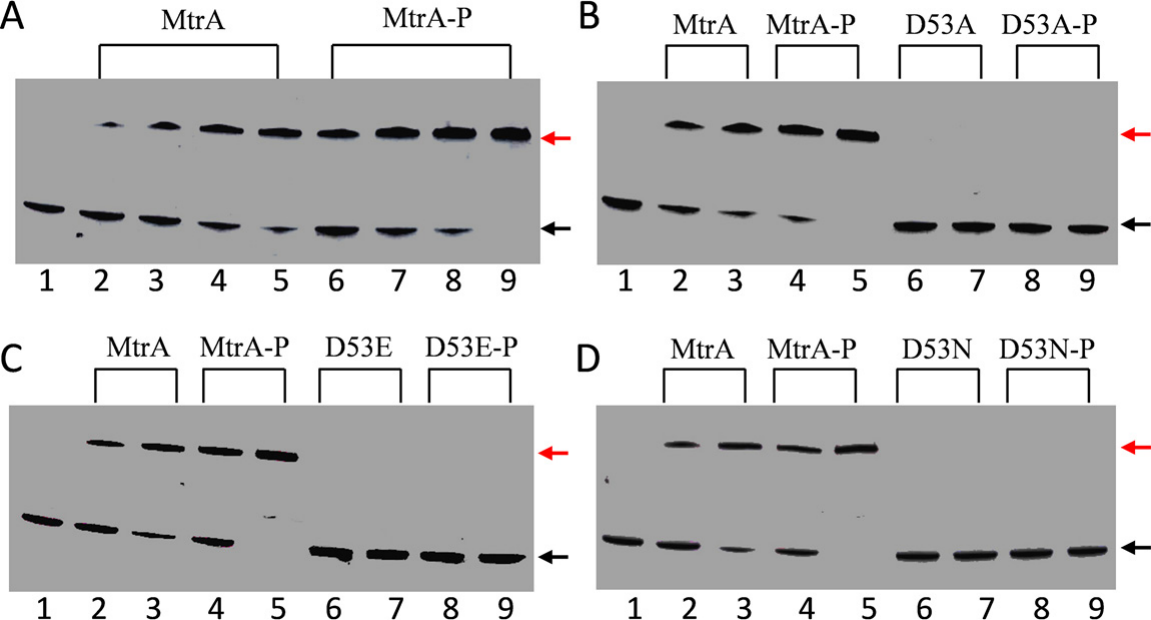
Effects of phosphorylation treatment on binding of wild-type MtrA and its variants to a target promoter. Protein binding to the *ureA* promoter probe with and without *in vitro* protein phosphorylation was compared by EMSA. (A) MtrA (lanes 2 to 5) and MtrA-P (phosphorylated MtrA; lanes 6 to 9) were incubated with the probe. Reactions were carried out with the addition of no MtrA (lanes 1) or 0.6 μM (lanes 2 and 6), 1.2 μM (lanes 3 and 7), 1.8 μM (lanes 4 and 8), or 2.4 μM (lanes 5 and 9) of unphosphorylated or phosphorylated wild-type MtrA. (B to D) Comparison of the binding of MtrA and its variants with and without *in vitro* phosphorylation treatment. MtrA (lanes 2 and 3), MtrA-P (lanes 4 and 5), MtrA variant (lanes 6 and 7), and phosphorylated MtrA variant (lanes 8 and 9) were incubated with the *ureA* promoter probe. Reactions were carried out with the addition of no MtrA (lanes 1) or 1.2 μM (lanes 2, 4, 6, and 8) or 2.4 μM (lanes 3, 5, 7, and 9) of MtrA or its variants. MtrA and its variants were incubated with 50 mM acetyl phosphate for 30 min at 30°C in 50 mM Tris-Cl (pH 8.0) and 5 mM MgCl_2_ before performing the EMSAs. Red and black arrows indicate the positions of the shifted and free probes, respectively.

We also investigated phosphorylation of the wild-type MtrA protein and its variants (Fig. 2). *In vitro* phosphorylation of wild-type MtrA protein enhanced its binding to the tested probe (Fig. 2A); however, after the same treatment, no shifting was detected for any of the MtrA variants under the same conditions (Fig. 2B to D), indicating that the wild-type MtrA was phosphorylation competent, whereas the MtrA variants were phosphorylation defective.

### Mutation of the D53 codon does not affect gene expression.

To determine if mutations at the codon for the phosphorylation site impacted the expression of *mtrA* in S. venezuelae, we used an S. venezuelae MtrA deletion mutant to engineer complemented strains that expressed FLAG-tagged versions of the wild-type (C-Δ*mtrA*_SVE_FLAG) or altered MtrA proteins (C-Δ*mtrA*_SVE_D53AFLAG, C-Δ*mtrA*_SVE_D53EFLAG, and C-Δ*mtrA*_SVE_D53NFLAG). The wild-type strain (10712) and the engineered strains were cultured on solid YBP medium, and cellular lysates were extracted at 18 h and 36 h of growth. Western blot analysis probing the lysates with anti-FLAG antibody showed that the altered forms of MtrA accumulated to levels comparable to that of the wild-type MtrA (Fig. S3), indicating that mutation of the D53 codon did not change the MtrA protein levels and that these MtrA variants were stable *in vivo*.

### Replacement of D53 abrogates the binding activity of MtrA *in vivo*.

**FIG 3:**
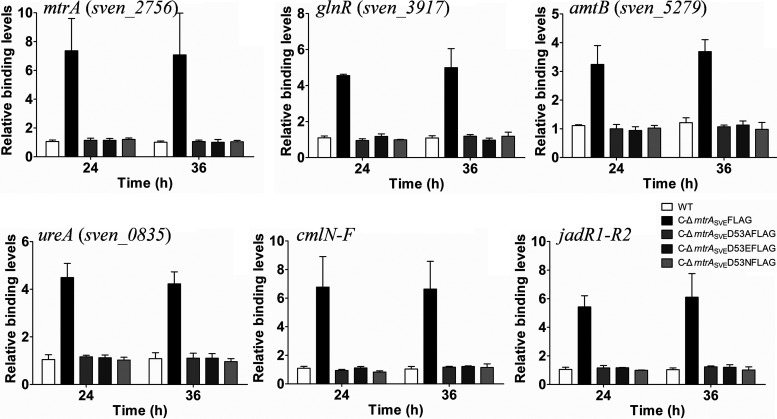
Comparison of the binding levels of MtrA and its variants to MtrA target sites *in vivo*. ChIP-qPCR analysis of the binding of MtrA and its mutants to the promoters of target genes in cultures grown on YBP. Analysis was performed using the wild-type strain 10712 (WT), C-Δ*mtrA_SVE_*FLAG, C-Δ*mtrA_SVE_*D53AFLAG, C-Δ*mtrA_SVE_*D53EFLAG, and C-Δ*mtrA_SVE_*D53NFLAG grown for the indicated times. The *y* axis shows the binding levels of MtrA-FLAG and MtrA-FLAG variants in these strains relative to background levels. Results are the mean values (±standard deviations [SD]) from triplet biological experiments and were determined by PCR analysis of target sequences recovered by treatment with anti-FLAG antibody.

Although the altered forms of MtrA accumulated similarly to the wild-type protein (Fig. S3), it was not known whether the MtrA variants still recognized MtrA sites *in vivo.* As MtrA-FLAG does function *in vivo* (18), we performed chromatin immunoprecipitation (ChIP)-quantitative PCR (qPCR) to quantify the binding levels of the FLAG-tagged MtrA variants to known MtrA sites. The ChIP-treated samples were prepared from cultures of the wild-type strain 10712 and engineered strains grown for 24 h and 36 h, and qPCR was performed to determine the relative amounts of MtrA target sequences recovered following immunoprecipitation of bound proteins with an anti-FLAG antibody (Fig. 3). Binding levels ranging from 1.0 to 1.21 were detected for the six tested genes in strain 10712, which does not express FLAG, indicating a minimal background for antibody binding, as reported previously (18). However, binding levels of 7.4 ± 2.2 (mean ± standard deviation) and 7.1 ± 2.9 were measured for the MtrA site of *mtrA* at 24 and 36 h, respectively, in C-Δ*mtrA*_SVE_FLAG. High levels of MtrA binding were also detected for the MtrA sites of *glnR* (24 h, 4.6 ± 0.1; 36 h, 4.99 ± 1.1), *amtB* (24 h, 3.2 ± 0.6; 36 h, 3.7 ± 0.4), *ureA* (24 h, 4.5 ± 0.6; 36 h, 4.0 ± 0.5), *cmlN-F* (24 h, 6.8 ± 2.1; 36 h, 6.6 ± 1.9), and *jadR1*-*R2* (24 h, 5.4 ± 0.7; 36 h, 6.1 ± 1.6), in agreement with a previous report (18). In contrast, binding levels of about 1, similar to the background level, were detected at these sites in C-Δ*mtrA*_SVE_D53AFLAG, C-Δ*mtrA*_SVE_D53EFLAG, and C-Δ*mtrA*_SVE_D53NFLAG, suggesting that MtrA variants with substitutions at D53 could not bind these MtrA sites *in vivo*.

### Replacement of D53 alters antibiotic production.

**FIG 4:**
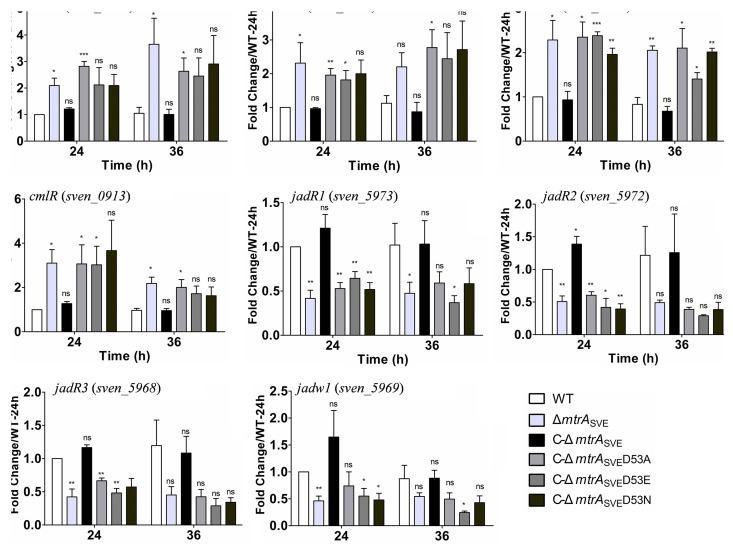
Transcriptional analysis of genes involved in antibiotic production in Streptomyces strains by real-time PCR. *Streptomyces* strains were cultured on solid YBP medium, and RNA samples from 10712 (WT), the Δ*mtrA*_SVE_ strain, C-Δ*mtrA_SVE_*, C-Δ*mtrA_SVE_*D53A, C-Δ*mtrA_SVE_*D53E, and C-Δ*mtrA_SVE_*D53N were isolated at the indicated times. The expression of *hrdB*, encoding the major sigma factor, was used as an internal control. For each gene, the expression level in the WT at the first time point was arbitrarily set to one. The *y* axes show the fold changes in expression in these strains over the levels in the WT at the first time point. Results are the mean values (± SD) from triplet biological experiments. Student’s *t* test was used for comparisons. *, *P* < 0.05; **, *P* < 0.01; ***, *P* < 0.005.

To investigate whether the D53 substitutions impacted the expression of antibiotic genes that are targets of MtrA (18), the expression levels of genes involved in antibiotic production were measured by real-time PCR in strains complemented with the mutated versions of *mtrA* (Fig. 4). Higher levels of expression were detected in the Δ*mtrA* mutant than in the WT and C-Δ*mtrA*_SVE_ for *cmlF*, *cmlI*, *cmlN*, and *cmlR* at the two time points tested, and the expression levels of these genes in C-Δ*mtrA*_SVE_D53A, C-Δ*mtrA*_SVE_D53E, and C-Δ*mtrA*_SVE_D53N were similar to those in the Δ*mtrA* strain, suggesting that the production of chloramphenicol (CHL) by these mutant strains might be similar to that in the Δ*mtrA* strain. On the other hand, the expression levels of *jadR1*, *jadR2*, *jadR3*, and *jadw1* in the Δ*mtrA* mutant were lower than those in the WT and C-Δ*mtrA*_SVE_, whereas the levels in C-Δ*mtrA*_SVE_D53A, C-Δ*mtrA*_SVE_D53E, and C-Δ*mtrA*_SVE_D53N were similar to the levels in the Δ*mtrA* strain, suggesting that jadomycin (JDM) production might be reduced in these mutant strains. In addition, our statistical analysis indicated that the expression levels of these antibiotic genes differed significantly between the MtrA D53 variant strains and Streptomyces venezuela ATCC 10712 at at least one time point.

**FIG 5:**
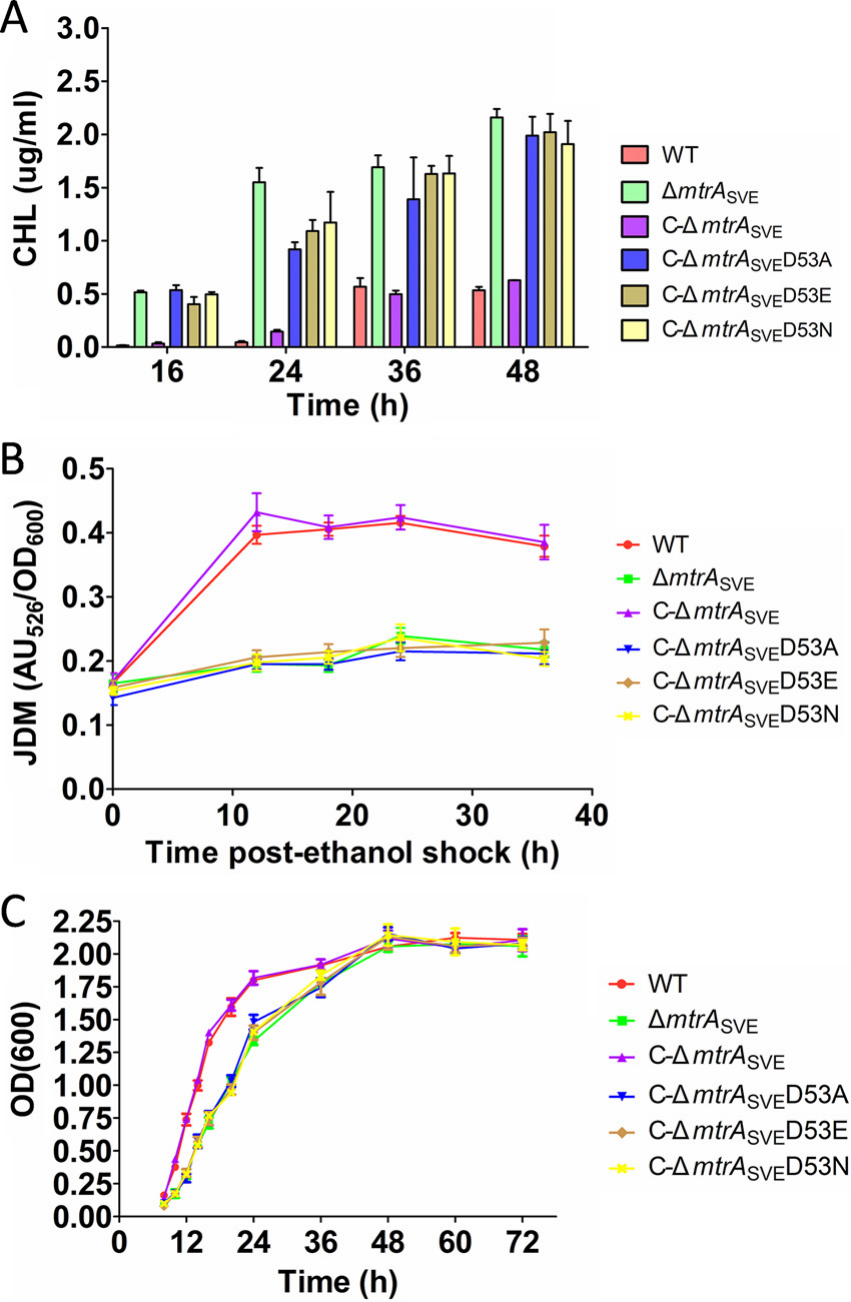
Comparison of CHL and JDM production by Streptomyces strains. (A) CHL levels were calibrated using a standard curve. (B) Production of JDM was calculated based on the units of absorbance of the culture supernatants at 526 nm normalized to the growth density at OD_600_ at each time point. (C) Growth curves of Streptomyces strains. The wild-type ATCC strain 10712 (WT), the *mtrA* deletion (Δ*mtrA_SVE_*) strain, *mtrA*-complemented strain C-Δ*mtrA_SVE_*, and strains complemented with mutated forms of *mtrA* (C-Δ*mtrA_SVE_*D53A, C-Δ*mtrA_SVE_*D53E, and C-Δ*mtrA_SVE_*D53N) were assessed. Results are the mean values (± SD) from triplet biological experiments.

The production of CHL and JDM was compared in strain 10712 (WT), the Δ*mtrA* mutant, and strains complemented with the wild-type *mtrA* (C-Δ*mtrA*_SVE_) or the mutated *mtrA* variants (C-Δ*mtrA*_SVE_D53A, C-Δ*mtrA*_SVE_D53E, and C-Δ*mtrA*_SVE_D53N). Compared to the levels in the WT and C-Δ*mtrA*_SVE_, CHL was overproduced by the Δ*mtrA* strain at the four time points tested (Fig. 5A), in agreement with a previous report (18). However, CHL was also overproduced by C-Δ*mtrA*_SVE_D53A, C-Δ*mtrA*_SVE_D53E, and C-Δ*mtrA*_SVE_D53N, with levels comparable to those of the Δ*mtrA* strain. In contrast to CHL production, the levels of JDM were much lower for the Δ*mtrA* strain than for the WT and C-Δ*mtrA*_SVE_, with the levels for C-Δ*mtrA*_SVE_D53A, C-Δ*mtrA*_SVE_D53E, and C-Δ*mtrA*_SVE_D53N being similar to that of the Δ*mtrA* strain (Fig. 5B). Collectively, these data indicated that complementation of the Δ*mtrA*_SVE_ strain with the MtrA D53 variants failed to restore production of CHL and JDM to wild-type levels.

### Impaired regulation of genes involved in primary metabolism in strains carrying MtrA D53 variants.

**FIG 6:**
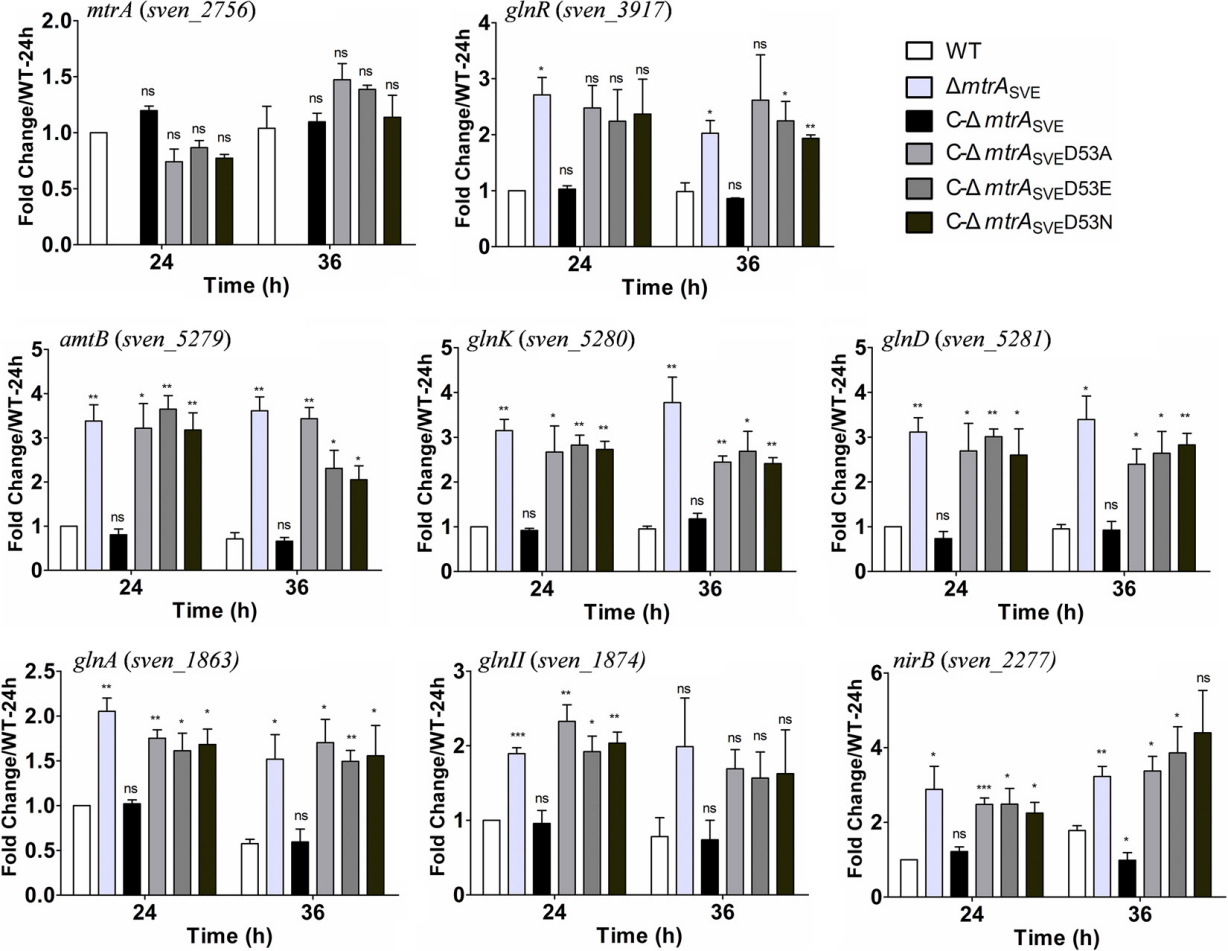
Transcriptional analysis of nitrogen metabolism genes in Streptomyces strains by real-time PCR. *Streptomyces* strains were cultured on solid YBP medium, and RNA samples from 10712 (WT), the Δ*mtrA*_SVE_ strain, C-Δ*mtrA_SVE_*, C-Δ*mtrA_SVE_*D53A, C-Δ*mtrA_SVE_*D53E, and C-Δ*mtrA_SVE_*D53N were isolated at the indicated times. The expression of *hrdB*, encoding the major sigma factor, was used as an internal control. For each gene, the expression level in the WT at the first time point was arbitrarily set to one. The *y* axes show the fold changes in expression in these strains over the levels in the WT at the first time point. Results are the mean values (± SD) from triplet biological experiments. Student’s *t* test was used for comparison. *, *P* < 0.05; **, *P* < 0.01; ***, *P* < 0.005.

Using the same RNA samples, we also investigated the impact of the D53 substitutions on the expression of nitrogen metabolism genes targeted by MtrA, as reported previously (23). The RNA samples used in this study were extracted from cell cultures grown on solid YBP, a nutrient-rich medium, and MtrA is known to repress nitrogen metabolism genes under such growth conditions (23). The expression of *mtrA* was verified in all of the strains complemented with either native MtrA or a D53 variant; however, as the primers were specific to the deleted *mtrA* sequence, no *mtrA* expression was detected in the Δ*mtrA*_SVE_ strain (Fig. 6). As expected, the expression levels of the nitrogen metabolism genes *amtB*, *glnII*, *glnA*, *glnD*, *glnK*, *glnR*, and *nirB* in the Δ*mtrA* strain were higher than the levels in the WT and C-Δ*mtrA*_SVE_, and the expression levels of these genes in C-Δ*mtrA*_SVE_D53A, C-Δ*mtrA*_SVE_D53E, and C-Δ*mtrA*_SVE_D53N were comparable to that in the Δ*mtrA* strain (Fig. 6), suggesting that the altered forms of MtrA failed to regulate the expression of nitrogen metabolism genes. Additionally, our statistical analysis indicated that the expression levels of nitrogen genes differed significantly between the MtrA D53 variant strains and M145 at at least one time point.

To evaluate the effects of the D53 mutations on cell growth, growth curves were compared for the WT, the Δ*mtrA* strain, C-Δ*mtrA*_SVE_, C-Δ*mtrA*_SVE_D53A, C-Δ*mtrA*_SVE_D53E, and C-Δ*mtrA*_SVE_D53N (Fig. 5C). The growth of the *mtrA* deletion strain was only slightly lower than that of the WT and C-Δ*mtrA*_SVE_ strains from 12 h to 36 h, and all three strains reached similar levels at the late growth stages, suggesting that deletion of MtrA did not have a major impact on cell growth. Notably, the growth curves of C-Δ*mtrA*_SVE_D53A, C-Δ*mtrA*_SVE_D53E, and C-Δ*mtrA*_SVE_D53N overlapped completely with that of the Δ*mtrA* strain, suggesting that these strains behaved similarly to the deletion mutant and excluding the possibility that the decreased JDM production was caused by reduced cell growth of these strains.

### *S. venezuelae* strains with D53 substitutions are defective in cellular development.

Deletion of *mtrA* in Streptomyces coelicolor M145 and S. venezuelae strain ISP5320 resulted in a conditional bald phenotype (21), suggesting that deletion of *mtrA* from S. venezuelae 10712 would most likely impact the cellular development of the resulting mutant strain. As expected, while the WT and the complemented strain C-Δ*mtrA*_SVE_ had already formed aerial hyphae by 24 h on MS medium or by 120 h on YBP, the Δ*mtrA*_SVE_ deletion strain was still bald (Fig. S4), in agreement with our previous report (21). Interestingly, all three strains complemented with mutated *mtrA* also appeared bald on both types of media (Fig. S4), indicating that the MtrA D53 variants were unable to restore the development of the Δ*mtrA*_SVE_ strain to the wild-type pattern.

## DISCUSSION

MtrA has been shown experimentally to be an important regulator in C. glutamicum (15, 28), in M. tuberculosis (8), and in Streptomyces species (21, 23). In this study, we show that the predicted phosphorylation site D53 of MtrA_SVE_ is required for binding and for the function of MtrA as a global regulator in S. venezuelae, as supported by the abrogated binding of MtrA sites by MtrA D53 variants both *in vitro* and *in vivo* and by the dysregulated expression of genes associated with antibiotic production and nitrogen metabolism and defective cellular development of strains expressing the altered versions of MtrA.

Previous studies had revealed the two residues D13 and D56 as critical for the phosphorylation of MtrA_TB_ (14). Similar to the D53 substitutions in MtrA_SVE_, substitutions D56E and D56N blocked the phosphorylation of MtrA_TB_ *in vitro* (14). However, in contrast to our D56E variant of MtrA_SVE_, MtrA_TB_D56E still retained the ability to bind its target genes *in vitro* (14), a difference that might be caused by strain-specific features. Although the replacement of D13 (D13A) blocked the phosphorylation of MtrA_TB_ *in vitro* (14), similar to our D56E alteration, MtrA_TB_D13A was still able to bind target sites. Growth defects of M. tuberculosis *mtrA* merodiploids overexpressing MtrA_TB_D13A suggested that MtrA_TB_D13A behaved like a constitutively active response regulator (14) and that it functioned in an unphosphorylated form. Although most response regulators are active in the phosphorylated form, exceptions have also been reported (29). Our data clearly show that MtrA_SVE_ requires phosphorylation for its activity. However, it will be interesting to investigate whether amino acid replacement at a residue corresponding to D13 of MtrA_TB_ or other amino acid changes at D53 of MtrA_SVE_ will create a constitutively active MtrA and how this would impact the phenotype of the resulting strains. In conclusion, this study reveals that D53 is essential for the regulatory role of MtrA in the genus Streptomyces.

## MATERIALS AND METHODS

### Bacterial strains, plasmids, primers, and growth conditions.

The strains and plasmids used in this study are listed in Table 1, and oligonucleotide primers are listed in Table 2. Streptomyces venezuelae 10712 (ATCC) was used as the wild-type (WT) strain. Streptomyces strains were grown at 30°C on maltose-yeast extract-malt extract (MYM) agar or liquid medium (30) for genetic manipulations, spore stock preparations, and chloramphenicol production. MS-G (soya flour glycerol) agar (31), YBP agar (32), and d-galactose–l-isoleucine liquid medium (33) were used for phenotype observation, RNA extraction, chromatin immunoprecipitation (ChIP) analysis, and determination of jadomycin production. Escherichia coli strain DH5α was utilized for plasmid construction and propagation, E. coli strain BL21(DE3) was used for heterologous protein expression, and E. coli strain ET12567(pUZ8002) (34) was used for transferring DNA from E. coli to Streptomyces species by conjugative transfer (35). E. coli strains were cultivated in LB liquid or agar medium with appropriate antibiotics (apramycin at 50 μg mL^−1^, hygromycin at 50 μg mL^−1^, ampicillin at 100 μg mL^−1^, chloramphenicol at 25 μg mL^−1^, kanamycin at 50 μg mL^−1^, or thiostrepton at 25 μg mL^−1^) when necessary.

**TABLE 1:** Bacterial strains and plasmids used in this study

Strain or plasmid	Description	Reference or source
Streptomyces venezuelae strains		
10712 (ATCC)	Wild type	41
Δ*mtrA* strain	*mtrA* deletion strain, Apr^r^	18
V-Δ*mtrA*_SVE_	Δ*mtrA* complemented with pMS82, Apr^r^ Hyg^r^	This study
C-Δ*mtrA*_SVE_	Δ*mtrA* complemented with pCom-mtrA, Apr^r^ Hyg^r^	18
C-Δ*mtrA*_SVE_D53A	Δ*mtrA* complemented with pCom-mtrAD53A, Apr^r^ Hyg^r^	This study
C-Δ*mtrA*_SVE_D53E	Δ*mtrA* complemented with pCom-mtrAD53E, Apr^r^ Hyg^r^	This study
C-Δ*mtrA*_SVE_D53N	Δ*mtrA* complemented with pCom-mtrAD53N, Apr^r^ Hyg^r^	This study
C-Δ*mtrA*_SVE_FLAG	Δ*mtrA* complemented with pMtrA-Flag, Apr^r^ Hyg^r^	This study
C-Δ*mtrA*_SVE_FLAGD53A	Δ*mtrA* complemented with pMtrAD53A-Flag, Apr^r^ Hyg^r^	This study
C-Δ*mtrA*_SVE_FLAGD53E	Δ*mtrA* complemented with pMtrAD53E-Flag, Apr^r^ Hyg^r^	This study
C-Δ*mtrA*_SVE_FLAGD53N	Δ*mtrA* complemented with pMtrAD53A-Flag, Apr^r^ Hyg^r^	This study
Escherichia coli strains		
DH5α	Used for general cloning	Shanghai Weidi
BL21 (DE3)	Used for protein expression, Cm^r^	Sangon
ET12567(pUZ8002)	Used for conjugation between E. coli and *Streptomyces*, contains helper plasmid pUZ8002, Cm^r^ Km^r^	35

Plasmids		
pMD18-T	General cloning vector, Amp^r^	Takara
pJTU1278	Shuttle vector for conjugation, Amp^r^	42
pMS82	*Streptomyces* integrative vector, Hyg^r^	43
pIJ773^*a*^	Plasmid used for cloning apramycin resistance genes, Apr^r^	Norwich, UK
pET15b	Plasmid used for protein expression in E. coli, Amp^r^	Novagen
pET28a	Plasmid used for protein expression in E. coli, Km^r^	Novagen
pCom-*mtrA*	pMS82 with 880-bp upstream sequence and 678-bp coding sequence of *mtrA*, Hyg^r^	This study
pCom-*mtrA*D53A	pMS82 with 880-bp upstream sequence and 678-bp coding sequence of *mtrA*D53A, Hyg^r^	This study
pCom-*mtrA*D53E	pMS82 with 880-bp upstream sequence and 678-bp coding sequence of *mtrA*D53E, Hyg^r^	This study
pCom-*mtrA*D53N	pMS82 with 880-bp upstream sequence and 678-bp coding sequence of *mtrA*D53N, Hyg^r^	This study
pMtrA-Flag	Plasmid with a 3× FLAG epitope inserted before the stop codon of *mtrA*, Hyg^r^	This study
pMtrAD53A-Flag	Plasmid with a 3× FLAG epitope inserted before the stop codon of *mtrA*D53A, Hyg^r^	This study
pMtrAD53E-Flag	Plasmid with a 3× FLAG epitope inserted before the stop codon of *mtrA*D53E, Hyg^r^	This study
pMtrAD53N-Flag	Plasmid with a 3× FLAG epitope inserted before the stop codon of *mtrA*D53N, Hyg^r^	This study
pEASY-BLUNT	General cloning vector, Amp^r^ Km^r^	Takara
pEX-*mtrA*	*mtrA* (of *S. venezuelae*) expression plasmid, Amp^r^ Cm^r^	18
pEX-*mtrA*_D53A_	*mtrA* D53A (of *S. venezuelae*) expression plasmid, Amp^r^ Cm^r^	This study
pEX-*mtrA*_D53E_	*mtrA* D53E (of *S. venezuelae*) expression plasmid, Amp^r^ Cm^r^	This study
pEX-*mtrA*_D53N_	*mtrA* D53N (of *S. venezuelae*) expression plasmid, Amp^r^ Cm^r^	This study

a The plasmid pIJ773 was from John Innes Centre.

**TABLE 2:** Primers used in this study

Purpose, primer	Sequence (5′–3′)^*a*^
*mtrA* and FLAG tag complementation	
MtrA Com-F	AAGCTTGTCGAGGGACTGCTCCAGGGCCTC (HindIII)
MtrA Com-R	AAGCTTGGGCTTCGGAGCAGCACTGCCTGT (HindIII)
D53A-F	GCCCTGATGCTGCCCGGAAGGGACGGCATCGAGGTGTGCCGGCT
D53A-R	CTTCCGGGCAGCATCAGGGCGAGCAGCACCAGGTCTGGCTTGGCCT
D53E-F	GAACTGATGCTGCCCGGAAGGGACGGCATCGAGGTGTGCCGGC
D53E-R	CTTCCGGGCAGCATCAGTTCGAGCAGCACCAGGTCTGGCTTGG
D53N-F	AACCTGATGCTGCCCGGAAGGGACGGCATCGAGGTGTGCCGGCTCAT
D53N-R	CTTCCGGGCAGCATCAGGTTGAGCAGCACCAGGTCTGGCTTGGCC
MtrA Flag-com-F	ACCCGGGGATCCTCTAGAGATTGTCGAGGGACTGCTCCAGGGCCTC
MtrA Flag-com-R	CCGCCTGAACCGCCTCCACCGCTCGGTCCCGCCTTGTACCCGACACCA
Linker-Flag-F	GGTGGAGGCGGTTCAGGCGGAGG
Linker-Flag-R	GCATGCCTGCAGGTCGACGATATCACTTGTCATCGTCATCCT
D53A-FLAG-F	GCTGCTCGCCCTGATGCTGCCCGGAA
D53A-FLAG-R	GCAGCATCAGGGCGAGCAGCACCAG
D53E-FLAG-F	CTGCTCGAACTGATGCTGCCCGGAAG
D53E-FLAG-R	GGCAGCATCAGTTCGAGCAGCACCAG
D53N-FLAG-F	GCTCAACCTGATGCTGCCCGGAAGGG
D53N-FLAG-R	CGGGCAGCATCAGGTTGAGCAGCA

Protein expression	
MtrA-Exp-F	CATATGAAGGGACGCGTTCTTGTCGTC (NdeI)
MtrA-Exp-R	AAGCTTGCTCGGTCCCGCCTTGTACCCG (HindIII)
MtrA-D53A-expF	GCCCTGATGCTGCCCGGAAGGGACGGC
MtrA-D53A-expR	CTTCCGGGCAGCATCAGGGCGAGCAGC
MtrA-D53E-expF	GAACTGATGCTGCCCGGAAGGGACG
MtrA-D53E-expR	CTTCCGGGCAGCATCAGTTCGAGCAGC
MtrA-D53N-expF	AACCTGATGCTGCCCGGAAGGGACGGC
MtrA-D53N-expR	CTTCCGGGCAGCATCAGGTTGAGCAGC

EMSAs	
SVEN0835 WT-F	CATGAGATCGCAAAGCCGCAGTTAACGAGCCGGAAAAACTTCCGCCCTAACGTGCAATG
SVEN0835 WT-R	CATTGCACGTTAGGGCGGAAGTTTTTCCGGCTCGTTAACTGCGGCTTTGCGATCTCATG
SVEN091516 WT-F	ACACTCCTTCTCCGCGCCGGGGGTGTCCAAGTCGTTAGACACGGCGTTCCGGGCGTTGC
SVEN091516 WT-R	GCAACGCCCGGAACGCCGTGTCTAACGACTTGGACACCCCCGGCGCGGAGAAGGAGTGT
SVEN1860 WT-F	GTTTCATCAGTGTTTGACCACCGGGTCACGCCCTGGTAACACCAGTCTGTGAGCCTGGT
SVEN1860 WT-R	ACCAGGCTCACAGACTGGTGTTACCAGGGCGTGACCCGGTGGTCAAACACTGATGAAAC
SVEN1863 WT-F	CCCACCCCGGTTAACTTCGACGAAACAATTGGGTCATGCTTGAGAAATCCCGTCTGCCT
SVEN1863 WT-R	AGGCAGACGGGATTTCTCAAGCATGACCCAATTGTTTCGTCGAAGTTAACCGGGGTGGG
SVEN1874 WT-F	AGGAAAGCTGAGTAACACGGGGTTCACATTCGGGCAACCGACGGGAAATCCCGTGTTGC
SVEN1874 WT-R	GCAACACGGGATTTCCCGTCGGTTGCCCGAATGTGAACCCCGTGTTACTCAGCTTTCCT
SVEN3917 WT-F	GCTCTCGCGCCCTGAGAGCTTTTGTTCATCCATCCGTAACAACGGTCGGAAAACGCAAA
SVEN3917 WT-R	TTTGCGTTTTCCGACCGTTGTTACGGATGGATGAACAAAAGCTCTCAGGGCGCGAGAGC
SVEN5279 WT-F	CCGGCCGTTCACGGTCGCGTAACACGCCCCACCCCTTCGTCACGGCTCCGAAACATCGA
SVEN5279 WT-R	TCGATGTTTCGGAGCCGTGACGAAGGGGTGGGGCGTGTTACGCGACCGTGAACGGCCGG
SVEN597273 WT-F	TGCGATTTCAATCATCACGATGTCAACTCCGTGTCAAATTTTCGTTGCACGACTCTCGG
SVEN597273 WT-R	CCGAGAGTCGTGCAACGAAAATTTGACACGGAGTTGACATCGTGATGATTGAAATCGCA
	
ChIP-qPCR analysis	
hrdB ChIP-qPCR F	GCCGAGGAAGGAATACAGCA
hrdB ChIP-qPCR R	AGGTCCTGGAGCATCTGGC
sven0835 Chip-qPCR F	GCAGTTAACGAGCCGGAAAA
sven0835 Chip-qPCR R	ACACCTTGTTGAACTGCGGA
sven0915/16 Chip-qPCR F	ATGGCTGTCTGGTGGGTCGTCA
sven0915/16 Chip-qPCR R	AAGCAGGGTCGTCAGAGGGTTA
sven2756 Chip-qPCR F	TGACATCCATGTCTGGCATCA
sven2756 Chip-qPCR R	GAACGCGTCCCTTCATATCG
sven3917 Chip-qPCR F	ACTGCCGGCTCTCGCGCCCTGA
sven3917 Chip-qPCR R	AGCCCCCGGGTCCTTTTGCGTT
sven5279 Chip-qPCR F	CGTTCACGGTCGCGTAACAC
sven5279 Chip-qPCR R	GTTTCCGTCGATGCCGCTC
sven5972/73 Chip-qPCR F	AGTCTCCCCGAAGTGACGCCGC
sven5972/73 Chip-qPCR R	ATTTGACACGGAGTTGACATCG

Real-time PCR analysis	
hrdB real-time F	CCAGATTCCGCCAACCCA
hrdB real-time R	CTTCGTCACGGTCGTCCTG
sven0835 real-time F	GAGCCCGCACGAACAGGAA
sven0835 real-time R	CAGGATGTGGGAGGTGAGGAG
sven0913 real-time F	CGACATCCACACCCTGCTG
sven0913 real-time R	ATCGGGGGGAACTCGGTCT
sven0915 real-time F	GCCGACGCCGAGGATGATG
sven0915 real-time R	CCTGATCCTCCTCGTGGC
sven0916 real-time F	ATGCCATTCGCCATCTACG
sven0916 real-time R	TGGAGGGACACGCCGAAAT
sven0924 real-time F	ACGAGAAATCCGAAGCCGC
sven0924 real-time R	GTAGGCGACCCAGCCCCAG
sven1863 real-time F	TCTACGACGAGACGGGCTACG
sven1863 real-time R	CAGGCGGTGGTACGAGTTCA
sven1874 real-time F	GGACCGTGTTCTCAAGCC
sven1874 real-time R	GTTGGACTCGTGCGGCGT
sven2277 real-time F	CCAGGATCGTGGTCCTCTGCGAG
sven2277 real-time R	GGGTCTTGCCCGAGAAGTACGAGGT
sven2756 real-time F	GTGCTGCGGGGTGAAGGGTT
sven2756 real-time R	CGTGTCGCTCTTGGCCGTGAG
sven3917 real-time F	TCGGGGGTGACGAAGCGG
sven3917 real-time R	CCAGGAGGTGTGGGGGTA
sven5279 real-time F	GCTGGTCCTCCGACTACG
sven5279 real-time R	GGTGAGGATGGCGAACAT
sven5280 real-time F	GGTCCCCAAGATCCGCAT
sven5280 real-time R	ACACCTTGCCGTCTCCGA
sven5281 real-time F	TCCTCCTGCTCCACGACG
sven5281 real-time R	GTGCGGACGGAGTGGTCG
sven5968 real-time F	ACGACCATCGGCGAGATCCTGGT
sven5968 real-time R	GCCAGATCTTCCTTGGAAGCAAAGTG
sven5969 real-time F	ACAGTTCGTCATGTGGGACC
sven5969 real-time R	CGGGTGATGTCGGAGCAGA
sven5972 real-time F	CCTGCTCGCCTCGGACAT
sven5972 real-time R	CAGCCATTCGCCGTTGTC
sven5973 real-time F	CGACACCCATGTGAGCAGCC
sven5973 real-time R	GCCGAAGCGGAAACCCAC

a Underlining indicates restriction enzyme sites.

### Construction of strains expressing MtrA variants.

The *mtrA* gene deletion (Δ*mtrA*_SVE_) strain and complementation strain C-Δ*mtrA_SVE_* (Δ*mtrA*_SVE_ complemented with wild-type *mtrA*) were obtained in our previous study (22). To construct strains expressing mutated *mtrA*, we used a previously reported strategy (36) to replace aspartic acid (D53) with alanine, glutamic acid, or asparagine, based on the complementation plasmid pCom-*mtrA* (22). The resulting plasmids were mobilized into the Δ*mtrA*_SVE_ strain by conjugation via E. coli ET12567(pUZ8002), generating the complemented strains C-Δ*mtrA*_SVE_D53A, C-Δ*mtrA*_SVE_D53E, and C-Δ*mtrA*_SVE_D53N. Screening and verification of the complemented strains were carried out according to previously described methods (34).

### Total RNA preparation, reverse transcription-PCR (RT-PCR), and real-time PCR.

Streptomyces strains (2 × 10^6^ spores) were inoculated onto YBP agar, and cultures were collected at multiple time points as indicated. Total RNA was isolated by using a SteadyPure universal RNA extraction kit (Accurate Biology), and cDNA was synthesized by using Evo M-MLV (Moloney murine leukemia virus) reverse transcriptase with the genomic DNA (gDNA) clean kit (Accurate Biology). Real-time PCR was performed by using a SYBR Green premix pro Taq HS mix (Accurate Biology) and the LightCycler 480II (Roche) as described previously (37). For calculation of the relative expression levels of tested genes, *hrdB* was used as the reference gene. The calculation method for the quantitative PCR (qPCR) data was described previously (37), and the data were obtained from three biological experiments.

### Expression and purification of MtrA and its variants.

Construction of plasmids expressing MtrA variants in E. coli was based on a previously described protocol (36). The resulting transgenic organisms were grown in LB medium at 37°C until the optical density at 600 nm (OD_600_) reached 0.6. To induce the overproduction of His-tagged MtrA and its variants, isopropyl-β-d-1-thiogalactopyranoside was added at a final concentration of 1 mM, followed by overnight cultivation at 16°C. These cultures were harvested, resuspended, and sonicated in lysis buffer containing 250 mM Tris-HCl (pH 8.0), 200 mM NaCl, and 20 mM imidazole. The supernatants were obtained after centrifugation (15,000 × *g* for 10 min at 4°C), loaded on Ni-nitrilotriacetic acid (NTA) columns (GE Healthcare), and eluted with binding buffer containing 250 mM imidazole. The eluted proteins were centrifuged in ultrafiltration tubes (Millipore), and the buffer was changed to storage buffer (50 mM Tris-HCl, 50 mM NaCl, pH 8.0). Purified proteins were assessed by sodium dodecyl sulfate-polyacrylamide gel electrophoresis (SDS-PAGE), and their concentrations were determined by using the Pierce bicinchoninic acid (BCA) protein assay kit (Thermo Scientific).

### EMSAs.

To phosphorylate MtrA and its variants, the proteins were incubated with 50 mM acetyl phosphate for 30 min at 30°C in 50 mM Tris-Cl (pH 8.0) and 5 mM MgCl_2_ (38). The proteins were treated without acetyl phosphate in parallel as negative controls for use in electrophoretic mobility shift assays (EMSAs). Complementary biotin-labeled primers (Table 2) were annealed to generate the EMSA probes, and the reaction conditions were as described previously (22). In brief, the DNA probes and proteins were mixed, incubated, and separated by 8% nondenaturing polyacrylamide gels. The gels were transferred and fixed on nylon membranes. Then, the membranes were blocked, washed, and processed as recommended (21). Finally, signals were detected by using the ECL Western blotting analysis system kit (GE Healthcare) followed by exposure to X-ray film or displayed using the myECL imager (Thermo Scientific). For some EMSAs (Fig. 1 and 2), after gel electrophoresis, the gels were stained with 4S red plus nucleic acid stain (Sangon) and imaged with a UV imager.

### Construction of engineered strains expressing FLAG-tagged MtrA.

To construct *Streptomyces* strains expressing the altered versions of MtrA tagged with the FLAG epitope, plasmids pMtrAD53A-FLAG, pMtrAD53E-FLAG, and pMtrAD53N-FLAG were constructed following the previously described protocol (39) and based on the plasmid pMtrA-FLAG (18). Conjugative transfer, screening, and verification steps for the FLAG-tagged strains were carried out essentially as described previously (22).

### Cellular lysate extraction, SDS-PAGE, and Western blot analysis.

S. venezuelae strains expressing FLAG-tagged proteins were cultivated on YBP agar at 30°C for 18 h and 36 h, and the cultures were harvested and extracted using liquid nitrogen. These crude extracts were resuspended in 0.5 mL lysis buffer (50 mM Tris-HCl, 50 mM EDTA, pH 8.0). Protein concentration was determined by using the Pierce protein assay kit (Thermo Scientific), and extracts were stored at −80°C until analysis. Each sample was separated by SDS-PAGE using 12% polyacrylamide gels and transferred to Hybond ECL membranes (GE Amersham). The membranes were blocked with 5% fat-free milk at room temperature for 2 h, washed, and then incubated with anti-FLAG monoclonal antibody (1:3,000; Boster Biological Technology) at 4°C overnight (40). Then, the membranes were washed twice and incubated with the horseradish peroxidase (HRP)-conjugated goat anti-mouse IgG (H+L) secondary antibody (1:5,000; Boster Biological Technology) for 50 min at room temperature. The signal was detected and imaged as previously described (22).

### ChIP-qPCR.

S. venezuelae strains were cultured on YBP agar and harvested at indicated times. ChIP was performed essentially as described previously (23, 39). The immunoprecipitated and input DNA were eluted in nuclease-free water, quantified using a NanoDrop spectrophotometer (Thermo Scientific), and subjected to qPCR analysis. The qPCRs and calculation of the binding levels were performed as reported previously (23).

### Growth curve analysis and detection of antibiotic production in *S. venezuelae*.

The growth curves of Streptomyces strains were determined as described previously (20). Briefly, equal numbers of spores (2 × 10^6^) were inoculated onto YBP liquid medium, and the optical density at 600 nm was measured at indicated times. Three replicate biological experiments were performed. To detect chloramphenicol (CHL) production in S. venezuelae strains, samples were treated and tested essentially as described previously (20). A standard curve using 10-fold dilutions of CHL (0.01 μg mL^−1^, 0.1 μg mL^−1^, 1.0 μg mL^−1^, 10 μg mL^−1^, 100 μg mL^−1^, and 1,000 μg mL^−1^) was generated for the quantitative analysis. To analyze jadomycin (JDM) production, S. venezuelae strains were cultivated and prepared essentially as described previously (33). Streptomyces strains were precultured in MYM medium and transferred to d-galactose–l-isoleucine medium (pH 7.5), and then 4% (vol/vol) ethanol was added to induce JDM production. Sample levels were calculated using the absorbance at 526 nm normalized to an OD_600_ of 1.

### CD spectroscopic assays.

CD spectroscopic assays for MtrA and its variants were performed by using a J-1500 spectrometer (Jasco, Japan) at 25°C. The quality of these proteins was evaluated by SDS-PAGE, and a protein concentration of 6 μM was used for spectral detection in a buffer of 50 mM Tris HCl (pH 8.0) containing 50 mM NaCl. The spectra were analyzed from 250 to 200 nm at a scan speed of 100 nm/min with a bandwidth of 1 nm.

### Sequence alignment analysis and modeling.

Alignment of MtrA and its variants was performed using CLC sequence viewer 6.5.3 (CLC Bio A/S), and models of these proteins were built by using SWISS-MODEL and visualized using PyMOL.
