# Supplementary material for: Mutation of MtrA at the Predicted Phosphorylation Site Abrogates Its Role as a Global Regulator in Streptomyces venezuelae
Source: Microbiol Spectr. 2022 Mar 16;10(2):e02131-21. doi: 10.1128/spectrum.02131-21 (PMC9045223; doi:10.1128/spectrum.02131-21)
Supplement: SUPPLEMENTAL FILE 1 — Supplemental material. Download SPECTRUM02131-21_Supp_1_seq8.pdf, PDF file, 0.6 MB [file spectrum02131-21_supp_1_seq8.pdf]

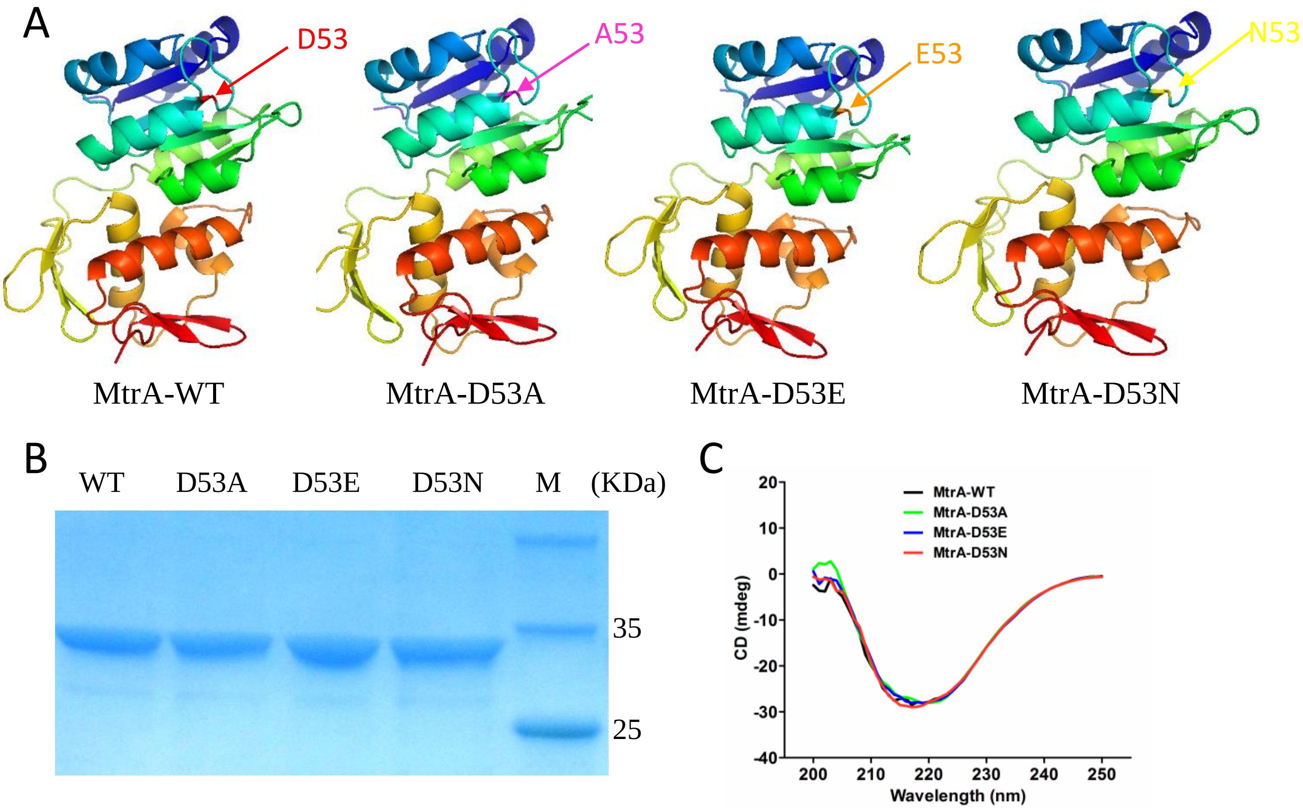

Figure S1. *In silico* and *in vitro* analysis of the wild-type and altered MtrA proteins from *S. venezuelae*. (A) The predicted tertiary structures of the wild-type MtrA (WT) and MtrA with substitutions at D53 (D53A, D53E, or D53N). The amino acid sequence of $MtrA_{TB}$ was used as the primary sequence for modelling with SWISS-MODEL software. (B) SDS-PAGE analysis of wild-type MtrA (WT) and its variants, heterologously expressed in *E. coli*. (C) CD spectra of wild-type MtrA and its variants. CD, circular-dichroism; mdeg, millidegrees.

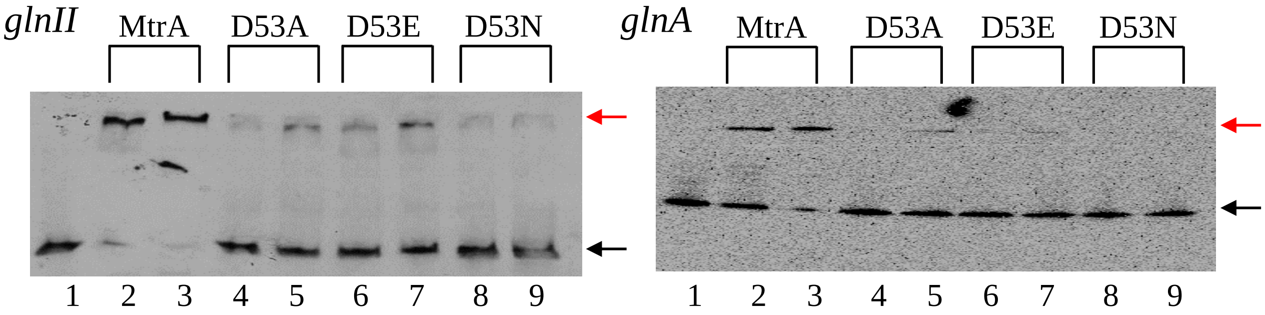

Figure S2. Comparison of the binding of wild-type MtrA and its variants to target sites by EMSA. Probes were used with (lanes 2 and 3) MtrA, (lanes 4 and 5) MtrAD53A, (lanes 6 and 7) MtrAD53E, or (lanes 8 and 9) MtrAD53N. Reactions were carried out with the addition of no MtrA (lanes 1), 4.7 μM (lanes 2, 4, 6, 8), or 8.3 μM (lanes 3, 5, 7, 9) MtrA or its variants. Red and black arrows indicate the positions of the shifted and free probes, respectively.

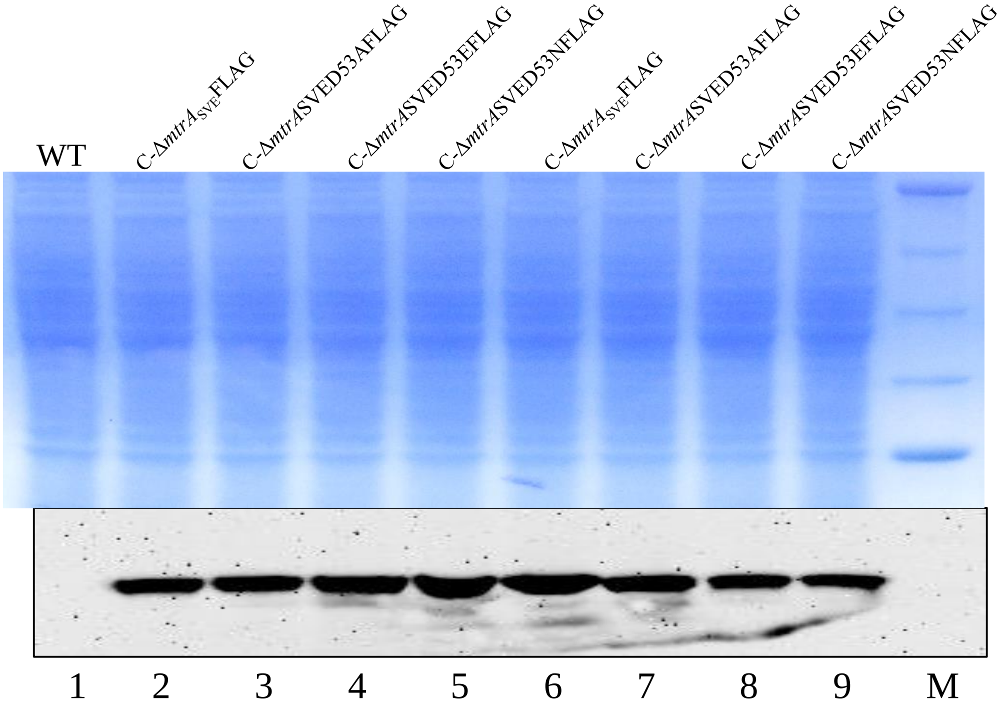

Figure S3. Western blot analysis probed with anti-FLAG antibody using 10 μg total cellular lysates extracted at 18 h (lanes 2-5) and 36 h (lanes 6-9) from mycelia of indicated strains grown on YBP. Upper image shows SDS-PAGE analysis of cellular lysates. M, marker lane.

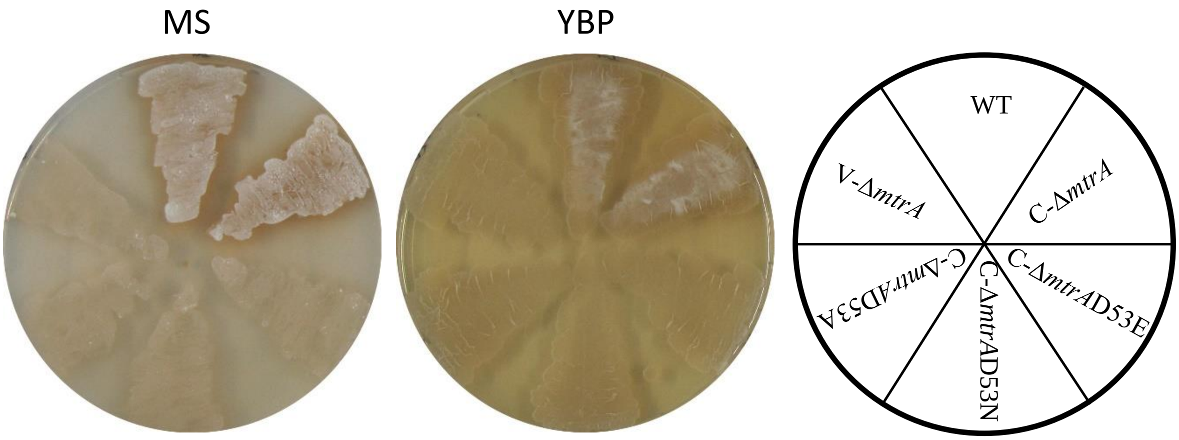

Figure S4. Phenotypic analysis of *S. venezuelae* strains expressing MtrA variants. Strain 10712 (WT), the deletion mutant Δ*mtrA*$_{SVE}$ containing the vector (V-Δ*mtrA*), and strains complemented with wild-type (C-Δ*mtrA*$_{SVE}$) or MtrA variants were grown on MS (24 h) and YBP (120 h) agar plates.
